# Using co‐design to develop a tool for shared goal‐setting with parents in speech and language therapy

**DOI:** 10.1111/1460-6984.12753

**Published:** 2022-07-20

**Authors:** Ingrid Singer, Inge S. Klatte, Rosa de Vries, Remko van der Lugt, Ellen Gerrits

**Affiliations:** ^1^ HU University of Applied Sciences Utrecht the Netherlands; ^2^ Utrecht Institute of Linguistics OTS Utrecht University Utrecht the Netherlands

**Keywords:** assessment, children, developmental language disorder, outcome, parents, speech and language therapists

## Abstract

**Background:**

Despite the compelling case for engaging parents in speech and language therapy, research indicates that speech and language therapists (SLTs) currently have a leading role in the goal‐setting process of therapy for children with developmental language disorder (DLD). Therefore, we set out to develop a tool that aims to support the dialogue between SLTs and parents and enhance shared decision‐making about children's communicative participation goals. We used co‐design techniques with SLT–practitioners to include their perspectives throughout the design process. Although co‐design has been used for some years in healthcare research, it is still a relatively new research methodology in the field of speech and language therapy.

**Aims:**

To provide a detailed description of the co‐design process that led to the development of a physical artefact that can support SLTs to engage parents of children with DLD in collaborative goal‐setting.

**Methods & Procedures:**

The Design Council's Double Diamond model was used to develop a tool in co‐design, together with eight SLTs, who participated in all stages of the development process. Usability was tested in actual goal‐setting conversations between four SLTs and 11 parents of a child with DLD resulting in stepwise improvements. In addition, usability of the first and final prototypes was tested with five usability criteria that were rated on a 10‐point scale by 64 SLTs.

**Outcomes & Results:**

The co‐design process resulted in the development of a physical prototype of the tool called ‘ENGAGE’, consisting of a metal ‘tree trunk’ on which parents can stick magnetic ‘leaves’ containing potential participation goals for their child. The ‘tree’ shape represents a child's development and opportunities for growth. This first prototype received marks between 7.0 and 8.0 out of 10 on attractiveness, user‐friendliness, safety, functionality and affordability. After several iterations, there were significantly higher marks for attractiveness, user‐friendliness and safety in favour for the final prototype. Marks for functionality and affordability did not change significantly.

**Conclusions & Implications:**

As researchers we usually develop pen‐and‐paper tools, interview protocols, apps or questionnaires to support clinical practice. Including the SLTs’ perspectives in the design process resulted in a tree‐shaped physical artefact that, according to the SLTs, helps to order information and encourages and guides their dialogue with parents. We strongly advocate the inclusion of end‐users in developing innovative user‐centred tools for speech and language therapy and we hope that this will become widespread practice.

**WHAT THIS PAPER ADDS:**

**What is already known on the subject**

- Collaborative goal‐setting is at the heart of family‐centred speech and language therapy. However, research indicates that goal‐setting processes for children with DLD are currently predominantly therapist‐led, instead of family‐centred. Reasons for the lack of parental engagement are that effective communication with parents throughout the goal‐setting process appears to be complex, and parents are not always invited and supported to engage in this. We used co‐design to develop a tool that aims to support SLTs in their dialogue about therapy goals with parents.

**What this paper adds to existing knowledge**

- This paper provides an example of applying a co‐design approach for the development of a shared goal‐setting tool for SLTs and parents of young children with DLD. The co‐design approach enabled us to incorporate needs, experiences and ideas of SLTs in the design process. We report the four stages in the co‐design process from (1) discovering the needs, wants and desires of the people involved, (2) defining the problem that SLTs experience, (3) developing several solutions and selecting the best solution, and (4) developing and testing the prototype. The detailed description of this process can add to an understanding of the advantages and disadvantages of a design process that includes the perspective of end‐users. The result is a physical artefact representing a tree, which aims to support the conversation between SLTs and parents about a child's communicative participation. Items describing facets of communicative participation are printed on ‘leaves’ that can be hung on a tree trunk by parents. The tree shape is a positive metaphor for the growth and development of a child.

**What are the potential or actual clinical implications of this work?**

- This study describes how SLTs can be meaningfully involved as partners in a co‐design research approach. Incorporating experience from clinical practice was highly relevant since our study aimed to create a solution that would support goal‐setting and service delivery by SLTs. We want to show that it is inspiring and beneficial for SLTs to partner with researchers in innovation of their own clinical practice and provide examples of co‐design activities that illustrate the involvement and influence of end‐users in a design process. Including the perspective of SLTs in the development of a new tool to facilitate the dialogue between SLTs and parents of children with DLD regarding therapy goal‐setting is expected to add value and enhance its implementation in clinical practice.

## INTRODUCTION

Children with developmental language disorder (DLD) have language problems enduring into middle childhood and beyond, with a significant impact on everyday social interactions or educational progress (Bishop et al., 2017). DLD affects 7% of all 5‐year‐old children (Tomblin et al., 1997), which means that on average two children in every classroom have DLD. Intervention for children with DLD consists of speech and language therapy delivered by speech and language therapists (SLTs), often in collaboration with professionals in preschools and schools. Since the family is the child's primary source of strength and support, it is important to deliver interventions in partnership with parents. Partnership is characterized by mutual understanding, a respecting and trusting relationship, shared decision‐making, and processes that incorporate family beliefs, needs and preferences (An & Palisano, 2014). A strong partnership between therapist and parent is thought to improve the quality and impact of the services provided, because it helps parents and children to receive the care they need when they need it (Law et al., 2012). In addition, parental involvement is expected to lead to improved decision‐making (Stevens et al., 2013), which is in turn associated with a better parent–therapist relationship (Stacey et al., 2017), more parent engagement (Klatte et al., 2019), and with better intervention outcomes for children (Coulter & Collins, 2011; Haine‐Schlagel & Escobar et al., 2016; Roberts & Kaiser et al., 2011; Van Voorhis et al., 2013). Our study focused on parental involvement in goal‐setting, because shared goal‐setting connects the therapy process with the child's and parents’ personal perspective and their communicative home environment, thus leading to relevant intervention outcomes (Baylor & Darling‐White, 2020; Paul & Roth et al., 2011; Wilcox & Woods et al., 2011; Woods et al., 2011). Setting goals for communicative participation draws heavily on the client values and preferences aspect of the evidence‐based practice triangle (E3BP) (Dollaghan et al., 2007). Parents of the client are most knowledgeable about their families’ preferences and coping style, as well as their specific physical and social communication environment (Baylor & Darling‐White, 2020).

### Shared goal‐setting

Scobbie et al. (2011) have identified four components of a goal‐setting and action‐planning practice framework: (1) goal negotiation, (2) goal identification, (3) planning and (4) appraisal and feedback. In the goal‐negotiation stage, parents consider the current situation and identify the main problem(s) they want to address. In the goal‐identification stage, the problem is refined into a specific, challenging goal agreed by both parents and the SLT. In the planning stage, parents are involved in translating goals into timely action. Finally, in the appraisal and feedback stage, parents receive support and feedback from the therapist. In our study we focused on the first two stages in Scobbie's framework: (1) goal negotiation and (2) goal identification, because we think that establishing a dialogue between SLTs and parents is essential here. An example of goal negotiation and identification could be an SLT asking parents what they would like to see their child accomplish over the next 2 months of therapy. Parents may start with a goal that refers to development of language skills, such as for their child to use more words. The therapist can then probe deeper into parents’ underlying values. She may discover that the parents’ priority is to foster their child's independence. Then, through discussion, the parents and therapist can discover what independence means for a 3‐year‐old child. Parents may indicate that this involves a degree of autonomy, for example, being able to ask for a preferred play activity or toy. Next, the SLT can discuss which situations offer opportunities to develop the target behaviour and explain to parents what this behaviour would look like. She could also explain which levels of support can be offered to the child to scaffold the development of the behaviour. This conversation may result in an example goal such as: ‘In 2 months, Sam can tell his preschool teacher which familiar play activity he would like to engage in during free play time.’

Despite the positive impact of engaging parents in speech and language therapy, research indicates that goal‐setting processes are currently predominantly therapist‐led, instead of family‐centred (Roulstone, 2015; Watts Pappas & McLeod, 2009). This seems particularly problematic when the aim of therapy is to improve communicative participation. Parental engagement in the articulation of communicative participation goals is key because only parents can tell which situations are most relevant for their young child's life (Baylor & Darling White, 2020; Grootens‐Wiegers et al., 2017). Yet, effective communication with parents throughout the goal‐setting process appears to be complex (Øien et al., 2010). What contributes to the complexity is that for parents it may be difficult to articulate participation goals because they draw on values, hopes and priorities in life which are often not clearly defined (Elwyn & Vermunt et al., 2020). In addition, parents may not know right away what their desired level of involvement in therapy is, and thus in goal‐setting (Epstein & Gramling, 2013). This complexity can result in SLTs not actually inviting and supporting parents to engage in the decision‐making and goal‐setting process. At the same time, SLTs tend to overestimate the level of actual parental engagement (Watts Pappas et al., 2008). This suggests that SLTs may be unaware of their dominant position in the decision‐making process.

To support SLTs in their collaboration with parents of children with DLD, we set out to develop a tool that can support SLTs and parents in the goal‐negotiation and goal‐identification stages of the shared goal‐setting process (Scobbie et al., 2011). The tool should be able to assist SLTs in their dialogue with parents about their priorities and concerns, as well as in setting and evaluating specific goals for communicative participation, together with parents.

### Decision support aids

Decision support aids, such as shared goal‐setting tools, can facilitate the exchange of information in an open conversation between client and service provider (Alston et al., 2014). They aim to help the client making informed choices about healthcare that reflect their personal values and preferences (Elwyn et al., 2010). Decision support aids encourage parents’ active participation in healthcare decisions affecting their child and improve partnership between the parent and the SLT (Barry & Edgman‐Levitan, 2012; Coulter & Collins, 2011; Holmes‐Rovner et al., 2007). In addition, decision support aids are considered important vehicles to achieve better healthcare outcomes and higher client and provider satisfaction (Van der Weijden et al., 2012). Decision support aids can be classified in three categories, depending on the context of use: use during face‐to‐face encounters, independent use by the patient, and use during remote client‐to‐coach and peer‐to‐peer encounters. Our study focused on use during face‐to‐face encounters. This type of decision support aid typically displays a limited amount of information that can easily be shared across a desktop (Elwyn et al., 2010). It aims to support shared decision‐making by making options visible and by organizing information in a way that a patient can understand. These tools are designed to improve the decision process by promoting dialogue and helping the clinician to engage the patient in a discussion about preferences (Elwyn et al., 2010). Although decision support aids have been available since the early 1980s, evidence suggest that their implementation into routine practice has been limited (Gravel et al., 2006). Many different cognitive (e.g., lack of knowledge), affective (e.g., motivation), social (e.g., patient acceptance) and environmental (e.g., reimbursement) factors may act as barriers for implementation (Holmes‐Rovner et al., 2007; Michie et al., 2005).

Although standards for decision support aid development do not prescribe specific ways or frequencies with which users must be involved (Coulter et al., 2013; Witteman et al, 2015), adapting tools to the needs of those who use them is expected to support successful implementation of decision support aids (Coulter et al., 2013; Vaisson et al., 2021; Witteman et al., 2015). This means that optimizing feasibility of actual use in clinical practice cannot be achieved without the input of the users of a decision aid (Vaisson et al., 2021). Therefore, we chose to develop the tool for shared goal‐setting together with SLTs and with researchers having a design or SLT background, and subsequently testing its usability in real life conversations with parents.

### Co‐design

Co‐design refers to the collective creativity of designers and people not trained in design working together in a design development process (Sanders & Stappers et al., 2008). It can be used to address a specific problem by bringing together the views, input and competencies of different stakeholders using a range of tools and exercises to optimize collaboration. According to Steen et al. (2011) co‐design can be beneficial for users, projects and organizations. User benefits include a better fit between the innovation and the user needs, a better user experience, and higher satisfaction. Projects benefit because co‐design improves the creative process, the central problem is better defined, and the project is organized more efficiently or effectively. Finally, organizations benefit through an improved focus on user needs, more creativity, better interdisciplinary cooperation, and more capabilities and enthusiasm for innovation (Steen et al., 2011).

Co‐design is thought to impact on participants directly (Robert et al., 2015), as it facilitates their empowerment, foster trust, and develops their autonomy, self‐determination and choice (Bowen et al., 2013; Palmer et al., 2019). It can reshape professionals’ work and make a meaningful contribution to realizing family‐centred care (Østergaard et al., 2017). Furthermore, the impact of co‐design is thought to reach beyond those who are directly involved, and lead to improvements in healthcare service delivery for the whole patient community (Boyd et al., 2012). Systematic research indicates that the level of end‐user engagement influences the outcomes of service redesign: structural outcomes, such as enhanced care, service delivery and governance, are associated with high‐level (co‐design) engagement (Bombard et al., 2018). However, there are also challenges associated with a co‐design approach, such as differences in power between participants, commitment to the co‐design process in terms of time and energy, use of appropriate methods for collaborative gathering and interpreting of experiences, involvement of participants not only in the experience gathering stages but also in the design of improvements, and finally moving a project forward towards actual implementation and subsequent impact (Dimopoulos‐Bick et al., 2019). Unfortunately, research on the impact of co‐designed tools within healthcare settings is currently lacking, and within the field of SLT no co‐design studies were found. Since co‐design with end‐users appears to lead to more useful and positive outcomes, we chose to use this methodology in our study. We report this co‐design approach to illustrate the benefits and challenges of this approach in developing new tools or resources for speech and language therapy.

### Aim

The aim of this paper is to provide a detailed example of the co‐design process in which a shared goal‐setting tool was developed for speech and language therapy.

The content of the tool was developed prior to this co‐design project, in a Delphi Study with parents, young adults with a language disorder, SLTs, teachers and teaching assistants, child psychologists, clinical linguists and clinical researchers (*n* = 47) (Singer et al., 2020). This Delphi panel developed 36 items indicating communicative participation of 2–8‐year‐old children with language disorders. Examples of items are: ‘the child asks for an explanation when he/she does not understand someone’, or ‘the child tells a clear story about something it did’ (for the full list of items, see Singer et al., 2020). We could have stopped at this point, and the SLTs might use the items as a topic list for a dialogue with parents on goals for therapy. However, to optimize actual implementation in clinical practice we decided to use the list of items to create a ‘tool’, which at that point, could be anything from an app, leaflet, questionnaire, interview protocol, game, framework, etcetera, to a physical artefact, which was the result of co‐design together with SLT end‐users.

## METHOD

### Design

The present study is a case study in which we used a co‐design approach and actively involved SLT–practitioners to develop a tool that can support their dialogue with parents about goal‐setting.

**FIGURE 1:**
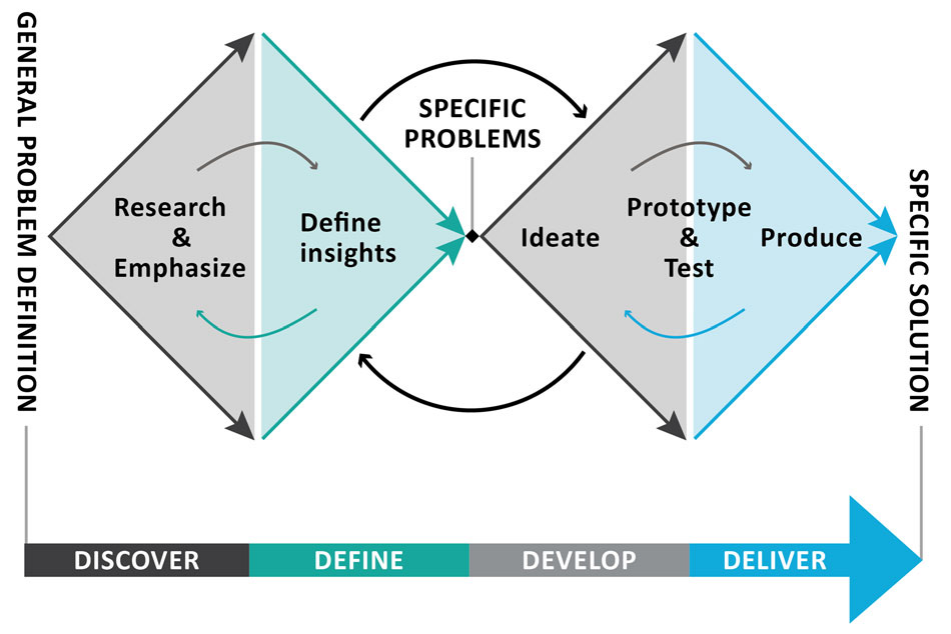
The Design Council's Double Diamond model (2007) [Colour figure can be viewed at wileyonlinelibrary.com]

The Design Council's Double Diamond approach guided our design process (Design Council, 2007). The model, developed to describe how the design process takes place in practice, consists of two diamonds (Figure 1) representing the two base points of the design process. Whilst the first diamond aims to ‘design the right thing’, the second diamond is directed to ‘design the thing right’. This process contains four stages: ‘Discover’, ‘Define’, ‘Develop’, and ‘Deliver’, starting with exploring an issue more widely or deeply and then taking focused decisions and actions, shifting from divergent thinking to convergent thinking. Although these stages appear to be successive steps, the real design process is not linear in nature. Rather, it can be seen as a dynamic and iterative process that allows designers to jump back and forth between the four stages in a way that complies with what is needed according to the current state of the design, and what is needed to advance the design most effectively (Dorst & Cross, 2001). We used co‐design research activities such as brain writing, dot voting, persona development, mind mapping, sorting tasks and more (e.g., Digital Society School, n.d.; Lewrick et al., 2020; Van ‘t Veer et al., 2020). The output of the activities was used as input for new activities or stages. For clarity, we have chosen to present these activities and the output in the results section of this paper.

**FIGURE 2:**
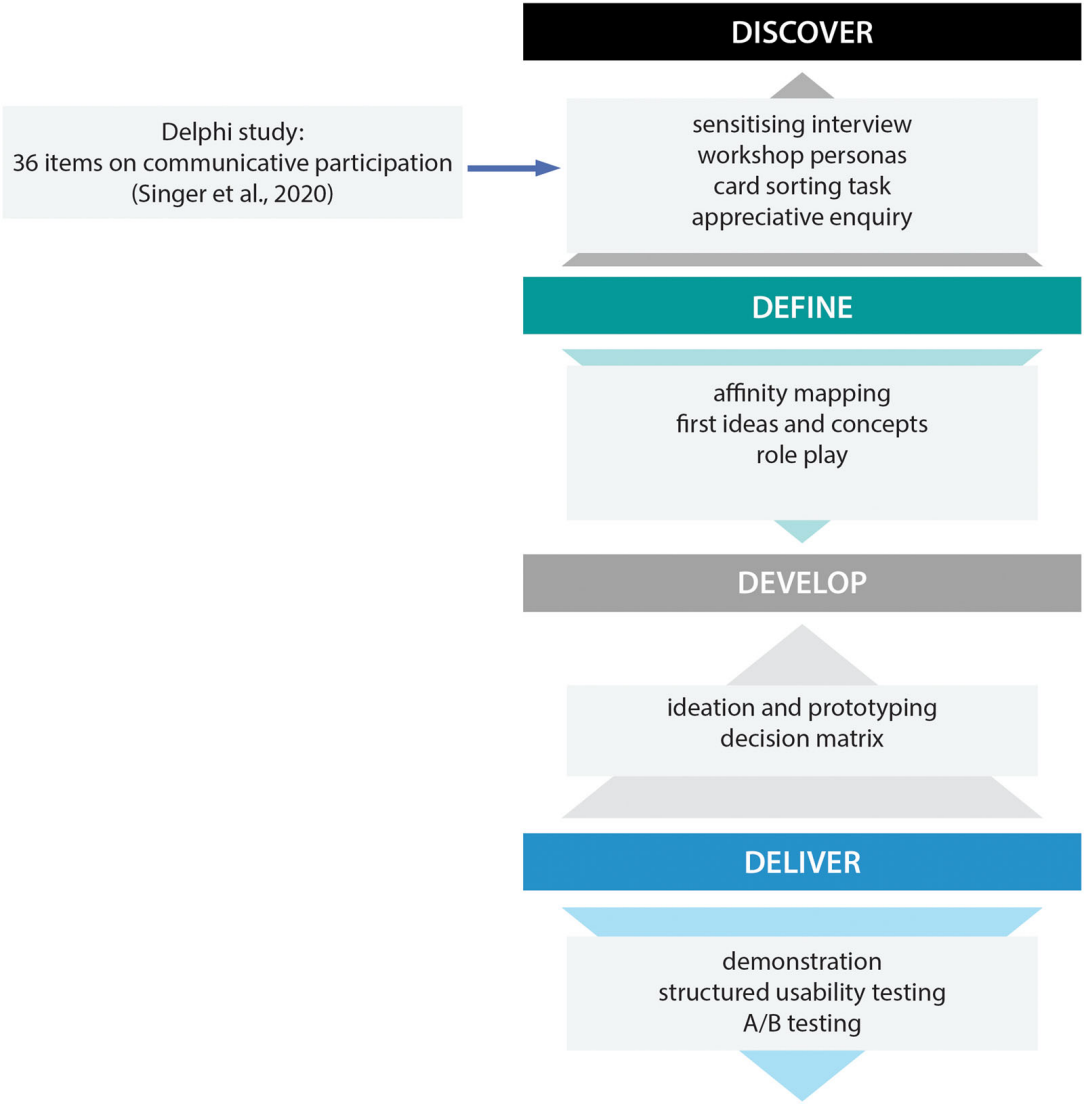
Stages of the Design Council's Double Diamond (2007) connected to activities in the present study [Colour figure can be viewed at wileyonlinelibrary.com]

Figure 2 displays which activities were planned in the various stages of the Double Diamond model.

### Participants

Participants were Dutch SLT–practitioners, SLT–researchers, co‐design researchers, co‐design students and parents of children with DLD (Table 1).

**TABLE 1:** Overview of participants in the various design stages

	***N***	**Discover**	**Define**	**Develop**	**Deliver**
SLT–researchers	3	✓	✓	✓	✓
Co‐design researchers	3	✓	✓	✓	✓
SLT–practitioners in co‐design workshops	8	✓	✓	✓	
Parents interviewed by SLTs before the first co‐design workshop	48	✓			
Co‐design students	4	✓			
SLT–practitioners in the usability study	4				✓
Parents in the usability study	11				✓
SLT–practitioners grading the first prototype	22				✓
SLT–practitioners grading the final prototype	44				✓
Total	145				

*Note*: Each row represents unique individuals who participated in one or more stages of the research project.

The project was initiated and coordinated by three SLT–researchers (authors IS, IK and EG), while the three co‐design researchers (RdV, RvdL and a third researcher who is not an author) were responsible for the planning and organization of the co‐design activities. Both SLT and co‐design researchers participated in all the stages of the project. The co‐design researchers had backgrounds in design and engineering, but their primary role in the project was that of researcher.

Eight SLT–practitioners participated as co‐designers in the Discover, Define and Develop stages. They were recruited via social media. Five of these eight SLT–practitioners worked in SLT practices in primary care, two in special education and one in a diagnostic centre. All SLT–practitioners worked with children with DLD and their parents. They had an average of 16 years of working experience as an SLT (range = 2–41 years). These eight SLT–practitioners each invited six parents of a child with DLD (in total 48) from their caseload to participate in a short interview. There were no selection criteria used. All SLTs gave their written informed consent to participate in the study. Parents gave verbal informed consent to the SLT to take their (anonymous) answers to the workshop.

The four co‐design students were recruited via a tutor of an international co‐design minor at our university. The students voluntarily selected our project for their co‐design assignment. They were majoring in communication and multimedia design at different universities in the Netherlands, South Korea and Ireland.

A new group of four SLT–practitioners was recruited via social media for the usability study in the Deliver stage. Of these four SLT–practitioners, three worked in primary care and one in special education. All SLT–practitioners worked with children with DLD and their parents. They had an average of 24 years of working experience as an SLT (range = 13–39 years) and gave their written informed consent to participate in the study.

Parents in the usability study were recruited via the SLT–practitioners and asked to use a prototype of the tool during their scheduled intake. Parents gave their written informed consent to participate in the study. Each SLT–practitioner tested prototypes with one to three parents, in two rounds. In total, 11 parents were involved in the usability testing. Because parents participated anonymously, demographic data on parents’ background was not collected. There were no selection criteria used.

Finally, two groups of SLT–practitioners graded the first (*n* = 22) and final (*n* = 42) prototype at two stakeholder meetings organized by the research group speech and language therapy. They were informed that their rating would be used for this study and handing their rating and feedback was voluntarily and anonymous.

This study was conducted following the principles of the Declaration of Helsinki (World Medical Association, 2013), and it was reviewed by the Internal Review Board of Health Sciences, HU University of Applied Sciences Utrecht, which concluded that the study is not subject to the Dutch Medical Research Involving Human Subjects Law (reference number 52_000_2017).

All data were processed anonymously and stored at a secured research server of our university with limited access, by authors IS, IK and EG.

## RESULTS

In this section we use the structure of the four stages of the design model to describe the various co‐design activities and their outcomes (Figure 2).

### Stage 1: Discover

The objective in this stage is to uncover users’ needs, which they may or may not be aware of and to discover who the users are, and which emotions guide their behaviour (Design Council, 2007). In our study the primary users were SLT–practitioners, and our primary focus was on their needs. Via the SLT–practitioners we also explored the views of the parents that are involved in their service delivery. Several co‐design activities were used which are described in detail in the paragraphs below.

#### Sensitizing interview

Sensitizers are appealing assignments to prepare and inspire participants for an upcoming co‐design workshop. This way, they already can start thinking about the subject of the session, doing some research or interviewing stakeholders (Sleeswijk Visser et al., 2005). To encourage the SLT–practitioners to explore aspects of their personal context before coming to the workshop, they were asked to have a short interview with at least five parents about their child's well‐being and the importance of certain values in life (e.g., health, relationships and education). Parents’ views on speech and language therapy and responsibilities in the therapy process were also incorporated. The questions were informed by research on parental perspectives of preferred outcomes for children with DLD (Law et al., 2015; Roulstone et al., 2012). The co‐design researchers developed visually attractive interview posters in A3 format, to guide SLT–practitioners and parents in the interview process and collection of responses. The interviews helped SLT–practitioners to build up an understanding of, and empathize with, parents’ needs, emotions, motivations and ways of thinking. After each interview, SLT–practitioners were instructed to take 10 min for self‐reflection on what was shared during the interview, to note their thoughts and observations on a dedicated space on the poster, and to bring the parents’ answers and their notes to the workshop.

#### Workshop personas

In the first co‐design workshop, SLT–practitioners (*n* = 8) were engaged in the development of personas. Personas are fictional ‘characters’ created in design research, with the intention to simplify communication and project decision‐making by a design team during the design process. They provide a context for designers of usage of an innovation (Lewrick et al., 2020: 97–102). In our study, personas were developed to help designers understand how diverse the parents are that SLTs encounter and to gain insight into how SLT–practitioners and parents’ may differ in their needs, experiences, behaviours and goals. The personas were used to help the designers recognize the diversity in parents that SLTs encounter when using the tool. In total, 48 interview posters containing parents’ answers and SLT–practitioners’ reflections on them, were brought to the co‐design workshop. SLT–practitioners were divided into two groups in which they talked about their interview posters. They reflected on similarities and differences between parents and constructed a mind map of the perceived differences between parents. After this assignment, the two groups presented their findings to each other. SLT–practitioners described eight experiences with parents from their SLT practice. These descriptions, together with the interview posters and workshop notes taken by the co‐design researchers, constituted the input for the creation of four personas that were given fictitious names (Lewrick et al., 2020: 97–102). These four personas are fictitious characters based on observations, interviews and notes that represent the diversity of parents that can be encountered within an SLT practice.

#### Card‐sorting task to categorize communicative participation items

The structure of the content of the tool was explored with a card‐sorting task (Wood & Wood, 2008). The objective was to learn how SLT–practitioners organize and categorize the content of the tool, the 36 items on communicative participation previously developed in the Delphi Study by Singer et al. (2020), for use in the next design steps so that the tool could be structured in a way meaningful for SLTs. The eight SLT–practitioners were randomly divided into three groups. In addition, the three SLT–researchers formed a group. Each group was handed 36 cards with one item from the Delphi Study (Singer et al., 2020) indicating communicative participation written on each card. The groups were asked to sort the items into one of four models familiar to many SLTs: Bloom and Lahey's (1978) model of language development, Gleason's model of language development (Gleason, 2005), the International Classification of Functioning, Disability and Health, Children and Youth Version (ICF‐CY; World Health Organization (WHO), 2007) and the United Nations Children's Fund's (UNICEF) (2009) developmental domains. Alternatively, groups could develop their own categories. Groups presented and explained their categorization after which all participants were asked to vote on their favourite categorization using dot stickers (dot‐voting; Tabaka et al., 2006). In total 33 stickers were used, and the number given for each categorization was counted. Subsequently, the four categorizations were presented and discussed at meetings with other experts such as the SLT research group, and SLT–lecturers of our university and several individual SLT–practitioners who were not involved in the previous workshop. This resulted in developing new categories, rewording categories and combining categories in a total of nine iterations.

#### Appreciative enquiry to develop design guidelines

Appreciative enquiry was used to enable the SLT–practitioners to develop design guidelines. Design guidelines are used across the co‐design cycle, whenever the team gets into situations where decisions must be made. At these critical points, design guidelines can support the team (Lewrick et al., 2020: 53–56). Appreciative enquiry was first developed in the field of organizational psychology as a method of generating innovative ideas about a topic of enquiry (Cooperrider & Whitney, 2005). The approach does not start with a predefined ‘problem’ that needs to be fully understood to remediate it but enables those involved in the process to focus on the ‘ideal’ situation instead. SLT–practitioners were asked to share their dreams about what an ideal tool would do, and how it would look, feel and work. One of the co‐design researchers facilitated this discussion, while two others took notes. After the workshop, the co‐design researchers translated their notes into seven ideas that could guide the design (Lewrick et al., 2020: 53–56).

#### Output Stage 1: Discover

**FIGURE 3:**
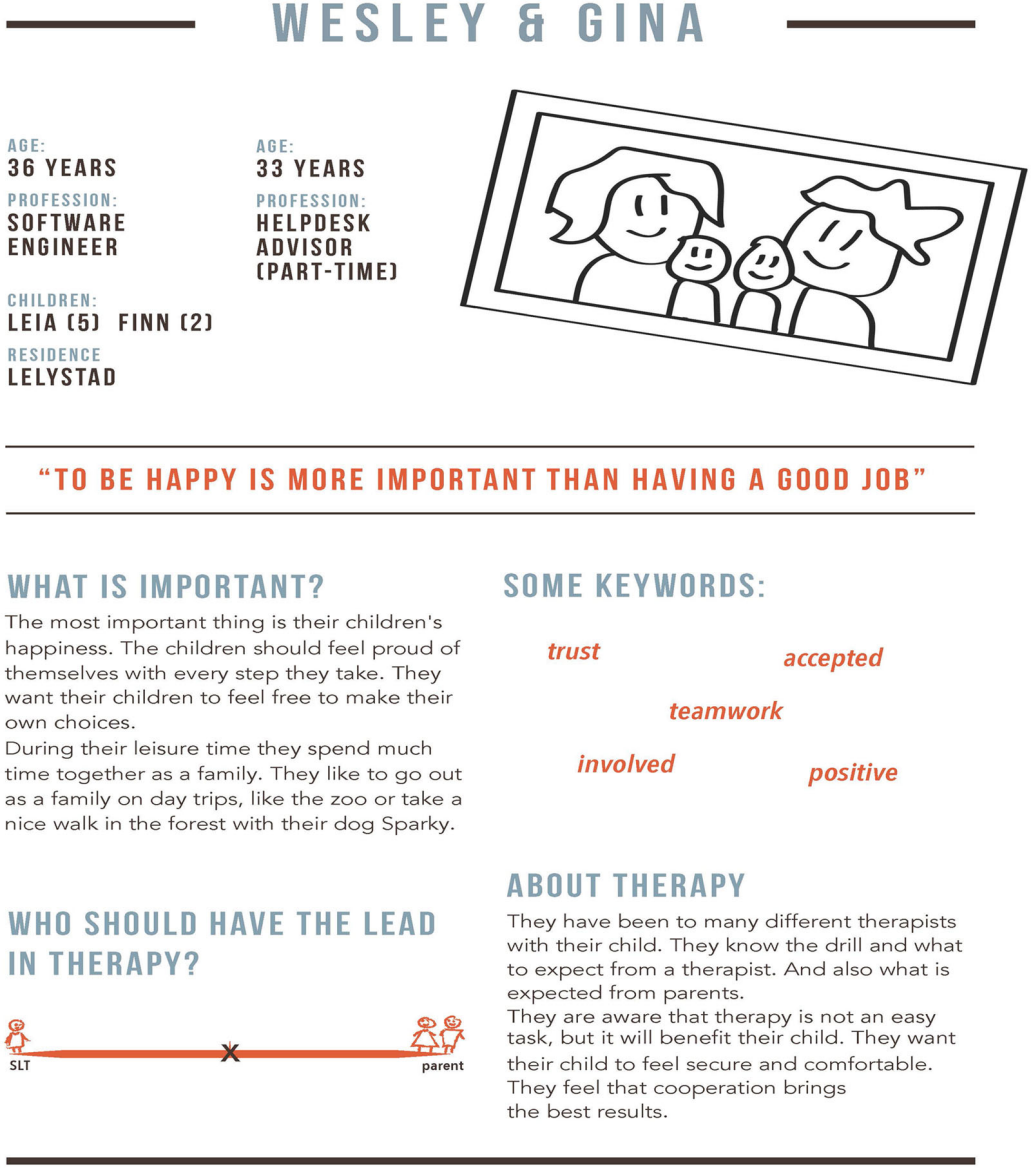
Example of a persona: ‘Wesley and Gina’ [Colour figure can be viewed at wileyonlinelibrary.com]

The output of the first co‐design activities were four personas with fictitious names ‘Wesley and Gina’, ‘Carine and Tim’, ‘Michaela’, and ‘Isaac and Miriam’. For an example of a persona, see Figure 3.

Table 2 shows the categories that three groups of SLT–practitioners and SLT–researchers developed to structure the 36 communicative participation items. The example models, such as ICF‐CY, that were provided by the research team where not used. Instead, each SLT group developed their own unique categorization. While groups 1 and 2 developed headings that could be interpreted without a specific order, groups 3 and 4 ordered the items from easy to complex. Furthermore, group 4 placed the items in a tree shape, with easy items in the root, moderate items in the trunk and difficult items in the branches. The result of the dot voting task is displayed in Table 3 and shows that the categorization by group 4 was favoured. For each categorization, one SLT–practitioner volunteered to explain what she saw as a key advantage of this solution. A quote from their explanation is displayed in Table 3.

**TABLE 2:** Categorization of the items on communicative participation

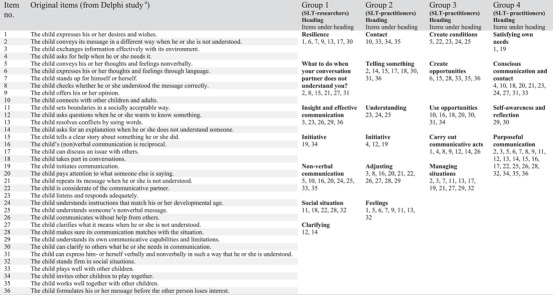

^a^ Singer et al., 2020

**TABLE 3:** Results of dot‐voting

**Group number**	**1**	**2**	**3**	**4**
Percentage of dot‐votes	21%	27%	15%	38%
Key advantage of a classification according to one of the participants	Resilience and non‐verbal communication are strong categories	Most categories are recognizable for parents	This categorization sorts items from ‘easy’ to ‘difficult’	The process of growth is visualized well by placing the cards in a tree

The SLT–researchers used the output in Tables 2 and 3, four categorizations, the outcomes of the dot‐voting and discussions with stakeholders to sort the 36 items of the tool into three categories named ‘communicative intention’ (four items), ‘understanding others’ (seven items) and ‘being understood’ (25 items) that were used in the further development of the tool.

**FIGURE 4:**
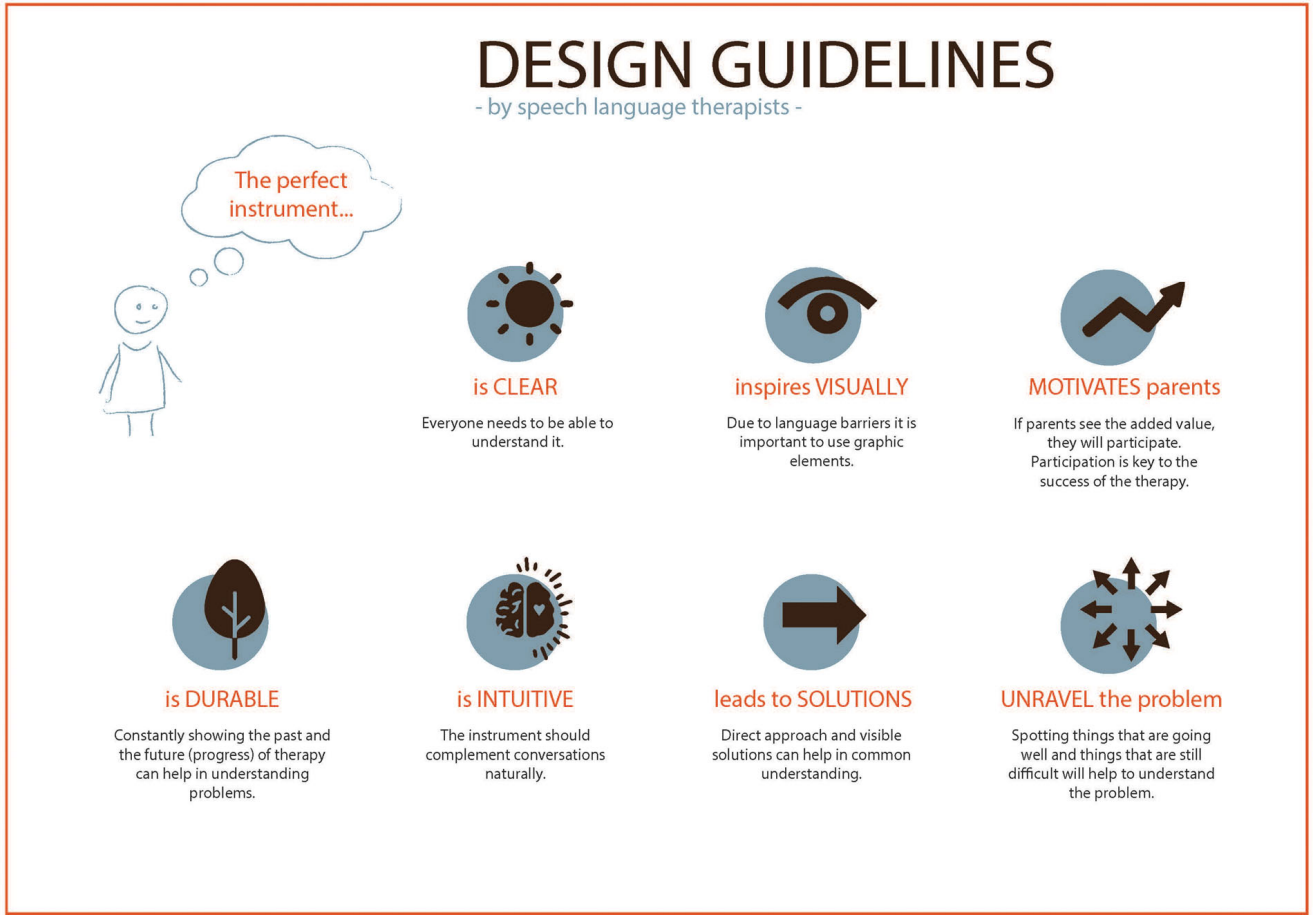
Design guidelines developed by the speech and language therapy (SLT) practitioners in the workshop [Colour figure can be viewed at wileyonlinelibrary.com]

The SLT–practitioners responded to the appreciative enquiry with ideas such as: ‘I would like to have a tool that motivates the parents to contribute to the conversation.’ The results from the appreciative enquiry were translated into seven design guidelines (Figure 4). In summary, the most important requirement according to SLT–practitioners was that the tool should have tangible, interactive and visual components to stimulate participation and engagement of the parent during the conversation with an SLT.

A team of co‐design students at our university used the personas, categories and items, and design guidelines to develop a first concept. The student team developed an ‘SLT collectible puzzle’, consisting of 36 pieces with the communicative participation items written on each piece (e.g., the child pays attention to what someone else is saying, the child communicates without help from others). The puzzle pieces had three colours that represented the three categories: communicative intention, understanding others and being understood. On the back of each puzzle piece there was room for notes, for example, a description of a goal or skill that the child could develop and more detailed and personalized assignments for a child. The idea was that parents could take a puzzle piece as a reminder of a particular language stimulating activity they can do at home, and that the child earns the puzzle piece as a reward when a goal is accomplished. According to the students, completing the puzzle illustrates children's growth and this will motivate parents to stay involved in therapy. This student‐concept was used as input for the next co‐design steps.

### Stage 2: Define

The objective in this stage is to state, explicitly and clearly, which problem keeps users from reaching their objective (Design Council, 2007). In our ‘Define’ stage, working mechanisms were explored and usability criteria were set up to inform development of prototypes.

The function of concept development and prototyping in this phase is for understanding the problem, whereas in the subsequent Develop phase, the focus shifts towards developing a fitting solution. Several co‐design activities were used, which are described in detail below. The activities were part of a workshop at our university and were facilitated by the co‐design researchers. The same eight SLTs as in Stage 1 participated, except for two SLTs who were unable to attend this second time. The workshop lasted for 4 h including several breaks.

#### Affinity mapping of product requirements

SLTs were asked to reflect on the ‘SLT collectible puzzle’ concept developed by the student team. While participants commented on the student‐concept, a co‐design researcher noted their positive and negative feedback (Van ‘t Veer et al., 2020: 188–191). This researcher categorized these comments together with the participants into an affinity map with five categories of product requirements. Affinity mapping is the collaborative process of organizing output from a discussion or brainstorming session into clusters or categories of similar items (Van ‘t Veer et al., 2020: 188–191).

#### First ideas and concepts

Six SLT–practitioners and two SLT–researchers individually developed a tangible concept, departing from the requirements just formulated. These concepts were early, sketchy and incomplete drafts intended to quickly illustrate potential working mechanisms (Lewrick et al., 2020: 199–202; Van ‘t Veer et al., 2020: 249–252). Participants used scrap materials, such as paper, wool, marbles, markers, containers and trays. The process was facilitated by the three members of the co‐design research group. After 20 min, a moment of reflection was built in to share individual results and to facilitate the combination of concepts into a maximum of three concepts in total. A total of 30 min were left to improve and strengthen the concepts in small teams.

#### Role play to identify working elements

Participants selected two concepts for exploration in terms of working elements during a role play with two SLTs: one in her own role and one in the fictitious role of a parent with characteristics matching one of the personas. The role play was used to further explore and validate the product requirements of the tool, while also allowing the SLTs to experience the solution and to interact with it. They experienced which mechanisms could work in the context of a conversation with a parent. The co‐design and SLT–researchers analysed the video recordings of the role plays to identify basic working elements for the solution (Lewrick et al., 2020: 199–202).

#### Output Stage 2: Define

All observational workshop data, such as photographs of the whiteboard with product requirements, videos of the role plays, and individual research journal notes were reviewed and discussed with the SLT–researchers and co‐design researchers. This resulted in a final set of product requirements for the tool: functionality, user‐friendliness, attractiveness, safety and affordability. Two important insights were gained from the concepting and role play activities. First, SLTs noted that handing the ‘parent’ a physical artefact resulted in the SLT to lean back and listen to the parent and thus seemed to facilitate parents in a dialogue with the SLT. Second, the SLTs playing the parent role predominantly talked about their child's skills and accomplishments, rather than about their experienced barriers and problems. These insights revealed that the biggest challenges in engaging parents in the goal‐setting process were to put parents in the lead and to focus on growth and development instead of focussing on barriers and problems.

### Stage 3: Develop

In this stage, as many ideas as possible are generated, prototyped, tested and iterated, all aiming at solving the users’ problem.

#### Ideation and prototyping

This stage started with a brainstorm to generate ideas (Lewrick et al., 2020: 151–154), building on insights from the earlier phases. We refer to the act of generating ideas, with the term ‘ideation’. When ideating, it is important to keep an open mind, and to retain, and build on, ideas that may seem too trivial and easy or too far‐fetched and complex (Isaksen et al., 2011). A multidisciplinary approach to ideation is encouraged, as it brings together varied perspectives which can lead to better outcomes (Van ‘t Veer et al., 2020).

Two members of the co‐design research group were also product and graphic designers, and they changed their roles during this stage from research facilitators to designers. Together with two SLT–researchers, ideas for prototypes were explored and developed. Two co‐design researchers and one SLT–researcher combined several ideas into three concepts and built a prototype for each concept.

#### Decision matrix

The three prototypes were presented and evaluated within the research team. To make a well‐grounded choice between the three concepts, they were evaluated against the design guidelines developed in Stage 1, using a decision matrix (Van ‘t Veer et al., 2020: 217–220). Consensus on the best prototype was reached through discussion between two co‐design researchers and two SLT–researchers.

#### Output Stage 3: Develop

**FIGURE 5:**
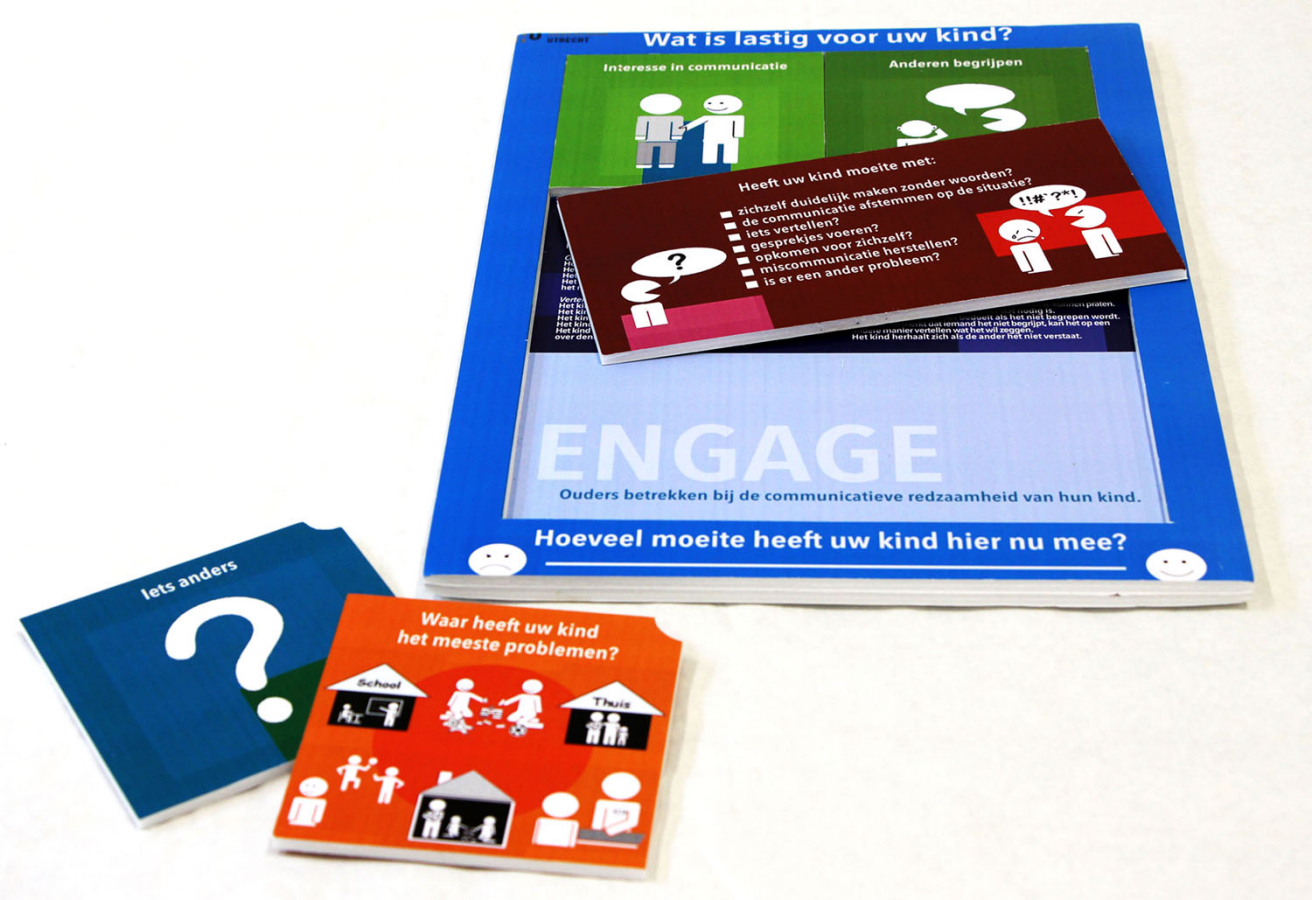
The ‘Jigsaw puzzle’ prototype [Colour figure can be viewed at wileyonlinelibrary.com]

**FIGURE 6:**
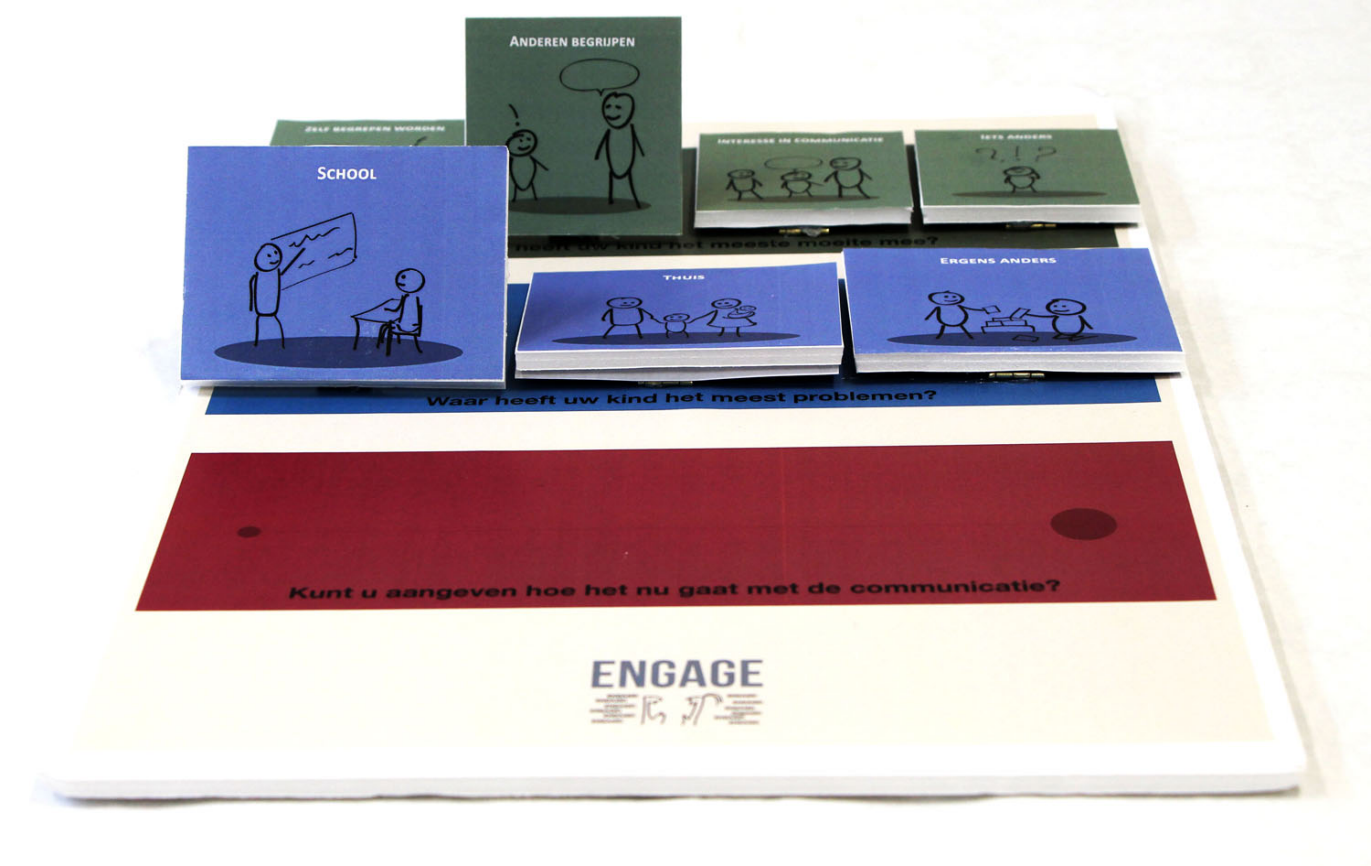
The ‘Guess who’ prototype [Colour figure can be viewed at wileyonlinelibrary.com]

**FIGURE 7:**
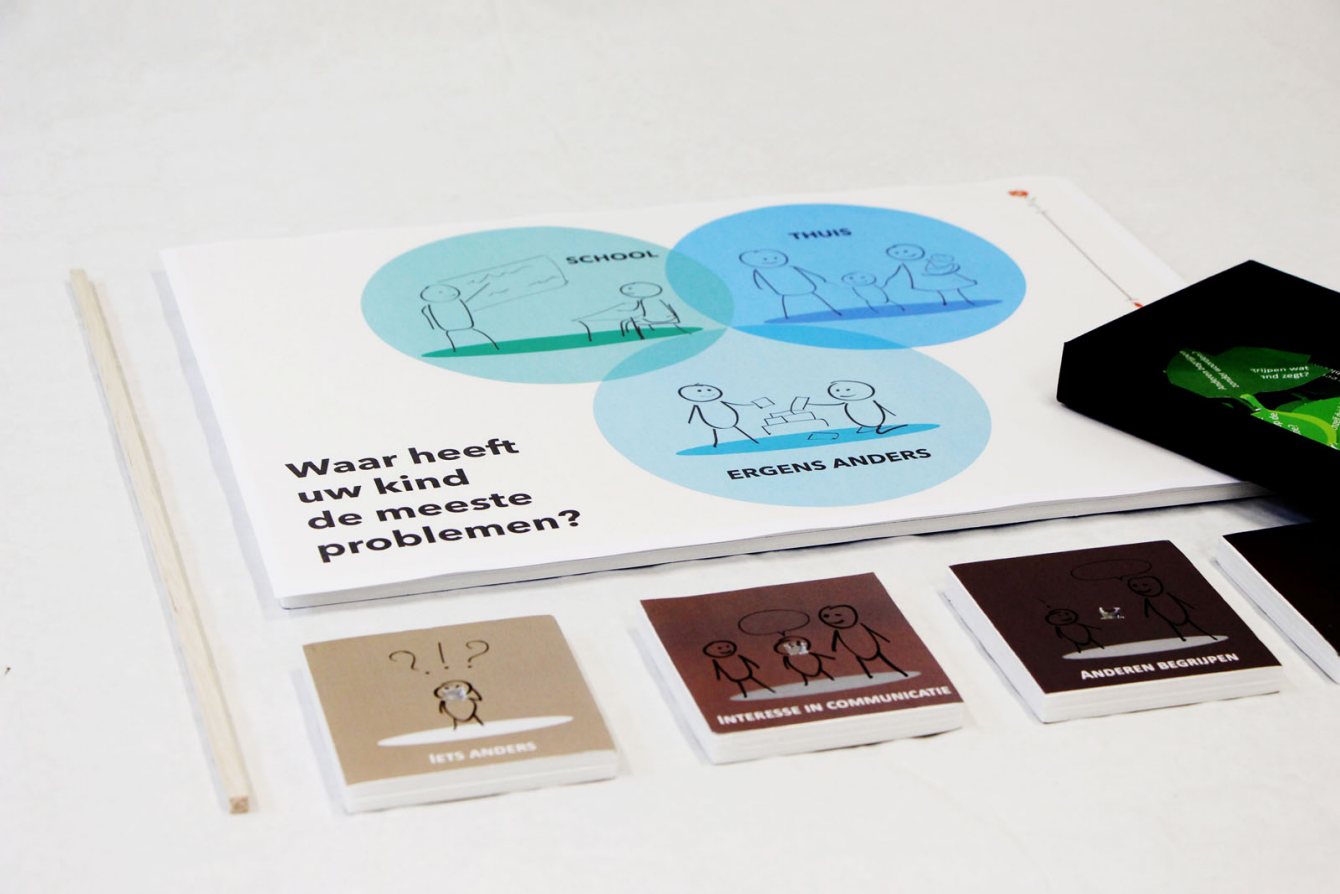
The ‘Tree’ prototype [Colour figure can be viewed at wileyonlinelibrary.com]

The first prototype was a board with five jigsaw puzzle pieces. Each puzzle piece had a red‐coloured side which indicated barriers in communicative participation, and a green‐coloured side for positive items (Figure 5). The second prototype was based on the game ‘Guess who?’ (Figure 6). In this prototype the user had to eliminate information to get to the core of the problem. The third prototype was a tree depicting the growth of the child's communicative abilities (Figure 7). Leaves could be placed high or low on a ‘tree trunk’ to indicate the performance in a communication skill.

The decision matrix is presented in Table 4. In both the ‘Jigsaw puzzle’ and ‘Guess who’ prototypes, a large amount of information was shown simultaneously, which made it harder to funnel the results. The jigsaw puzzle also contained too much text that was not supported by icons or images, which contradicted with the requirements of visual support. ‘Guess who’ was less intuitive than the other two prototypes; instead of getting more information during the use of the tool, the information had to be eliminated from an extensive amount of information to begin with. The ‘tree‐concept’ was evaluated as the best prototype, because it had an excellent match with the design guidelines. Overall, it was the most intuitive product, and the SLT–researchers thought it was inspiring, as the tree shape visualizes the concepts of growth and development. Another advantage was that the tool facilitated a structured and gradual way of sharing information in a conversation, and that pieces of information could be handed to parents, in order to elicit active participation.

**TABLE 4:** Decision matrix where prototypes are evaluated against design guidelines

**Design guidelines**	**Jigsaw puzzle**	**Guess who?**	**Tree**
The tool is clear	–	±	+
The tool inspires visually	±	±	+
The tool motivates parents	–	+	+
The tool is durable	–	–	+
The tool is intuitive	±	+	+
The tool leads to solutions	–	±	+
The tool dissects the problem	–	–	+

The research team evaluated the fit and function of the winning ‘tree’ prototype using the four personas. For example, we reasoned that the parents in our example persona, Gina and Wesley, who were described as very capable in expressing their concerns and needs, still might benefit from using the tool, because it marks the process of shared goal‐setting and decision‐making. For the SLT the expected advantage of using the tool was the opportunity to share observations in a dialogue with parents.

### Stage 4: Deliver

The last stage of the Double Diamond model is the delivery of the project, resulting in the finalization of the outcome, for example, a product or a service. This stage revolves around developing and testing the final concept, prior to actual production and implementation (Van ‘t Veer et al., 2020). In our deliver stage, we used the results of the structured usability testing to develop multiple iterations of the tree‐prototype (Lewrick et al., 2020: 229–232), and conducted an A/B test to verify whether the adaptations had been successful (Lewrick et al., 2020: 233–235).

#### Demonstration

**FIGURE 8:**
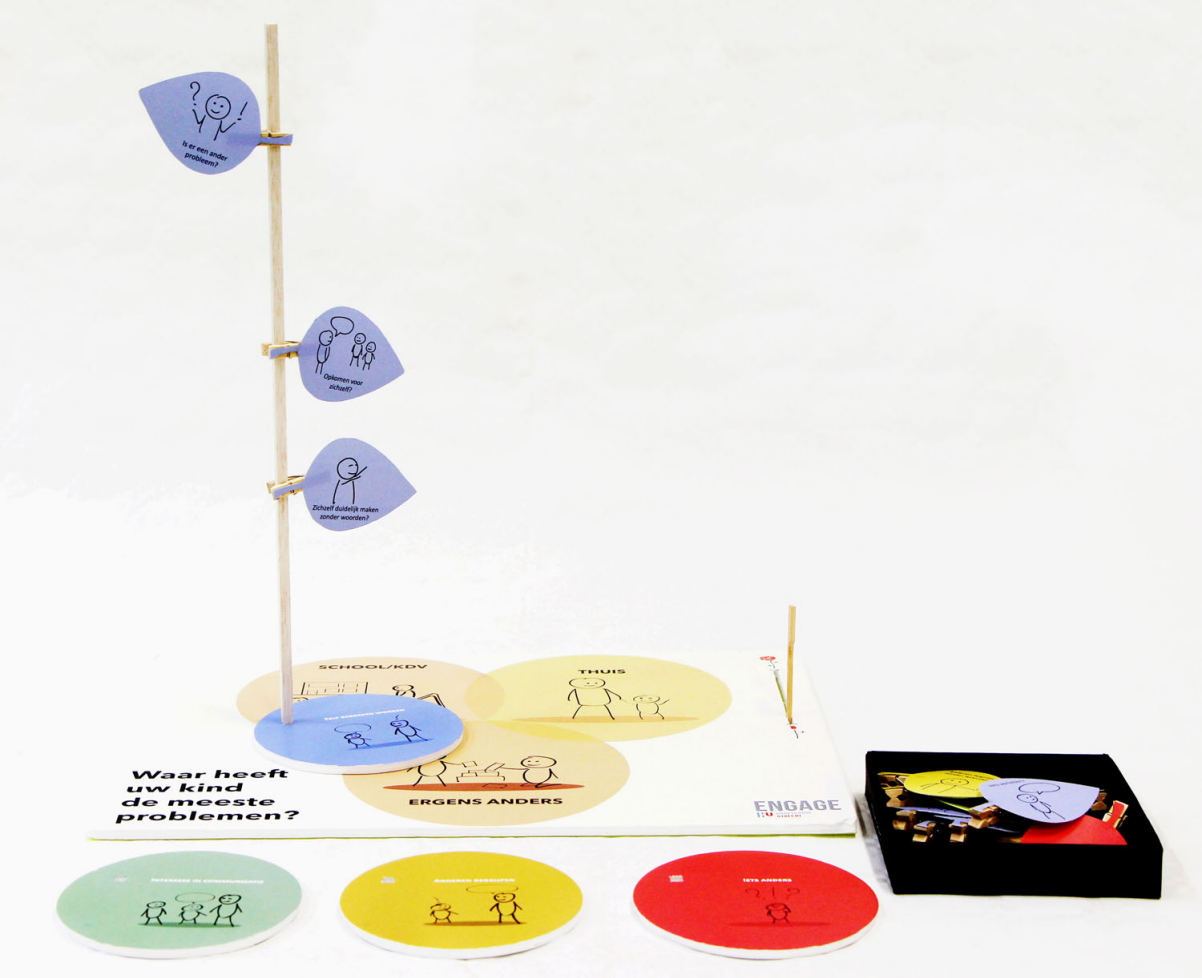
First iteration developed in the testing stage [Colour figure can be viewed at wileyonlinelibrary.com]

To receive feedback on the first tree prototype (Figure 7), it was demonstrated in a workshop on a continuing education symposium for SLTs. The participating SLTs had not been involved in previous stages of the present study. After a live demonstration of the tool, SLTs (*n* = 22) filled in a feedback form that included the product requirements as usability criteria. Comments and suggestions mentioned by more than one SLT were fed back to the co‐design researchers who adjusted the tool accordingly (Figure 8).

#### Structured usability testing

**FIGURE 9:**
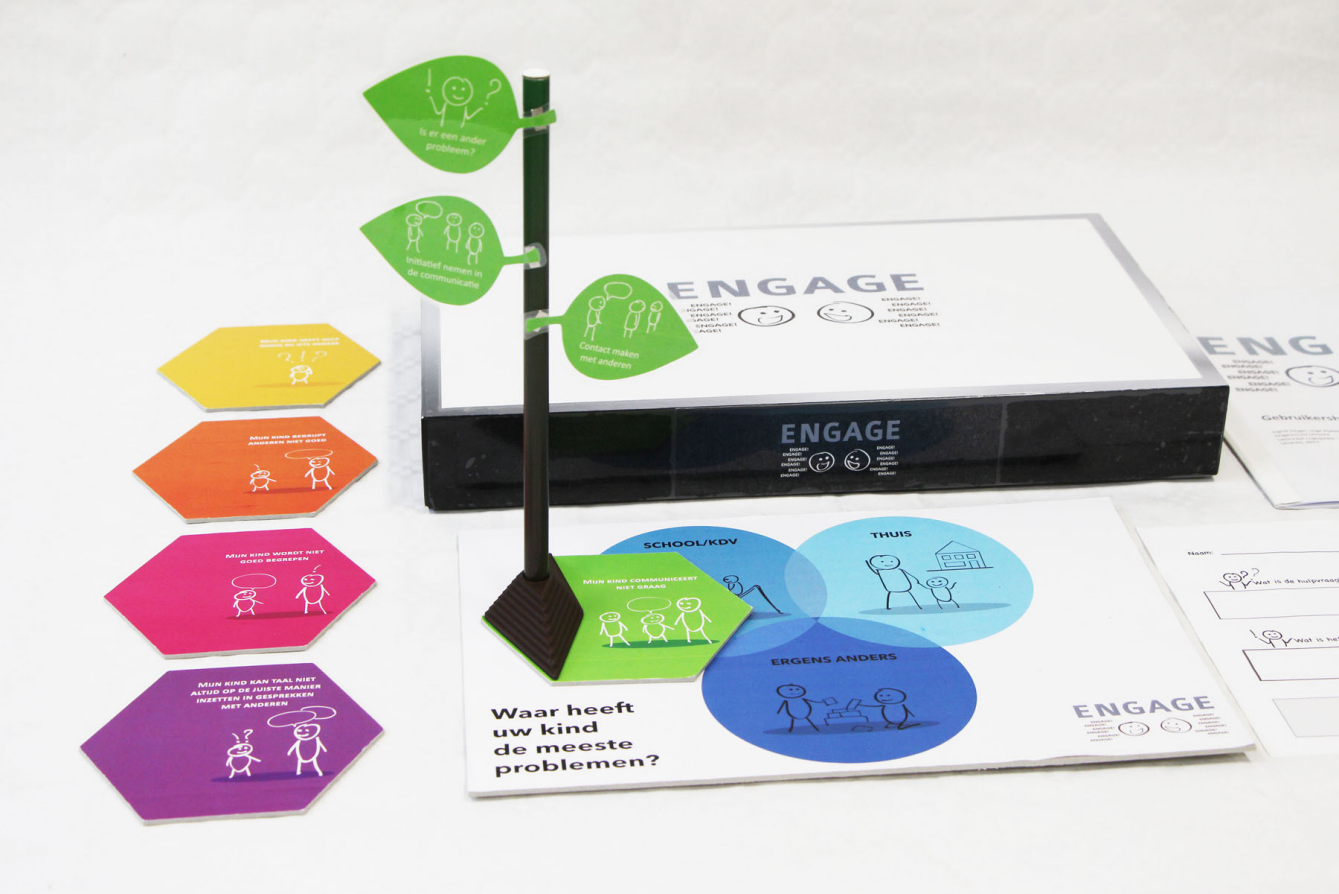
Second iteration developed in the testing stage [Colour figure can be viewed at wileyonlinelibrary.com]

**FIGURE 10:**
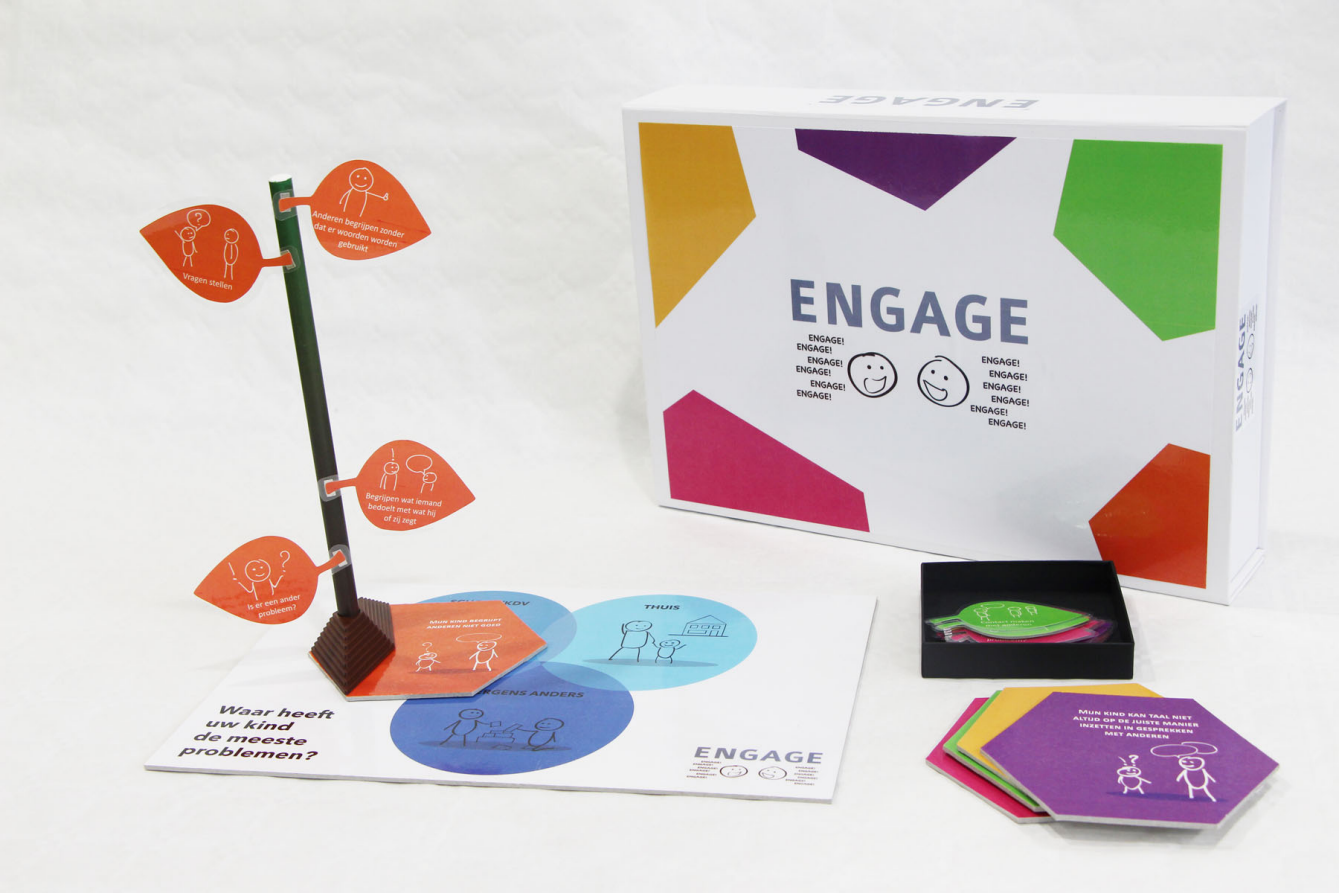
The final ‘Tree’ prototype developed in the testing stage [Colour figure can be viewed at wileyonlinelibrary.com]

Four SLTs invited parents to discuss their child's communicative participation problems. First, the SLTs prepared the conversation by reading a draft instruction manual, while commenting aloud on any vagueness in how the tool could be used. Their comments were used to improve the manual. Remaining questions form the SLTs were answered by the SLT–researchers. In the next step, three SLTs used the tool (Figure 8) together with five parents. SLTs’ findings were reported in a feedback form that included the product requirements as usability criteria. SLTs discussed their answers with an SLT–researcher. In addition, the SLT–researchers interviewed the parents about their experiences with the tool, focusing on the same criteria. After the first test round, the comments of the SLTs and parents, as well as parts of the video recordings of the conversations were fed back to the co‐design researchers who adjusted the tool (Figure 9), while the SLT–researchers adjusted the user manual and texts in the tool. The updated version of the prototype and manual was used in a second test round that was performed with the same procedure. The three participating SLTs invited six other parents to participate in this round and obtained their informed consent. The tool was adjusted again after this round of usability testing (Figure 10).

#### A/B testing

To evaluate whether the usability of the first (Figure 7) and the final prototype (Figure 10) had changed, an A/B test was conducted. A/B testing is a user‐experience research methodology wherein two versions, A and B, of a product are compared (Lewrick et al., 2020: 233–236). The first (A) and final prototype (B) were demonstrated at two separate occasions at a workshop of a continuing education symposium for SLTs. None of the attending SLTs had been involved in previous stages of the present study. Each SLT rated either the first (*n* = 22), or the final (*n* = 42) prototype on the five usability criteria using a 10‐point Likert scale, with a score of 1 indicating the worst possible performance and a score of 10 for the best possible performance. To test the tool for ‘affordability’, we asked participants to rate two different selling prices for the first and the final prototype. This way we wanted to determine whether an increased production value (i.e., robustness, level of detail and finishing) of a prototype was reflected in a higher perceived value by SLTs. Affordability was marked against a fictious selling price of €50 for the first prototype and €75 for the final prototype.

#### Output Stage 4: Deliver

Structured usability testing resulted in several iterations leading to a final prototype (Figure 10): a physical artefact that we called ‘ENGAGE’. It consists of a metal ‘tree trunk’, on which parents can stick selected ‘leaves’, with items representing relevant participation goals for their child. Parents place the trunk on a board with three circles referring to participation at ‘home’, ‘school/day care centre’ and ‘somewhere else’, to indicate in which context(s) their child needs support. Tree leaves that are placed higher in the tree represent the child's acquired competences, and tree leaves placed lower in the tree are potential goals for therapy. Together with the tool, a form was developed for writing down a personalized goal for communicative participation. On this form, parents can score a 10‐point Likert scale, indicating how well the child is performing on this goal at the start of a therapy period. Scoring can be repeated after working on that goal for some time. A higher score in an indication of progress.

SLTs commented on the prototypes concerned the colour scheme, the choice of materials, its safety and robustness, the clarity of categories of items, understandability of texts in the tool, the need for a form to write down and evaluate goals, and the comprehensiveness and coherence of the text in the manual for the SLT. Whilst the first prototype contained 36 items in three categories (communicative intention, understanding others, and being understood), the final version had 17 items and four categories (likes to communicate, understands others, is understood and uses language in conversations). Feedback on the complexity of the items resulted in items being reworded from C and B2 language levels into the less complex A2 or B1 Common European Framework of Reference for Languages language levels (CERF; Council of Europe, 2001). As a result of rewording the items, some were merged, reducing the total number of items in the tool from 36 to 17. The extra category ‘uses language in conversations’ was created, because SLTs thought that the category ‘is understood’ had too many items and the set of skills that it described was too broad. They suggested to create an extra category for complex language use in conversations. Based on their feedback we also added a separate information sheet with examples illustrating each item in the manual of the final version. Feedback from parents resulted in in changing the visual analogue scale for goal evaluation into a Likert scale, which parents found easier to understand and use, and adding space on the form for describing activities that they can do with their child to work towards the goals.

**FIGURE 11:**
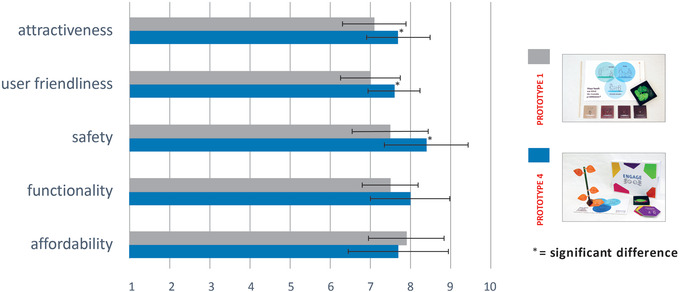
Speech and language therapy (SLT) practitioners ratings of the first and final prototype [Colour figure can be viewed at wileyonlinelibrary.com]

The first (Figure 7) and final (Figure 10) prototypes were rated using a 10‐point Likert scale (Figure 11). The first prototype received marks between 7.0 and 8.0 out of 10, while the final prototype received marks between 7.5 and 8.5 out of 10, indicating sufficient usability for both prototypes.

The A/B testing results were further analysed with independent‐samples *t*‐tests comparing the ratings of the first and final prototypes (Table 5). In summary, there were significant differences in the scores for *attractiveness, user friendliness* and *safety* in favour for the final prototype. Differences between marks for *functionality* and *affordability* were not significant.

**TABLE 5:** Results of an independent samples *t*‐test on usability ratings of the first and final prototypes

	**First prototype, mean; SD**	**Final prototype, mean; SD**	***t*‐test (d.f.), *p***
Attractiveness	7.1; 0.82	7.7; 0.78	*t*(61) = –2.87, *p* = 0.006^a^
User friendliness	7.0; 0.74	7.6; 0.67	*t*(60) = –3.26, *p* = 0.002^a^
Safety	7.5; 0.97	8.4; 1.07	*t*(53) = –2.95, *p* = 0.005^a^
Functionality	7.5; 0.68	8.0; 0.98	*t*(60) = –1.81, *p* = 0.075
Affordability	7.9; 0.93	7.7; 1.25	*t*(59) = 0.415, *p* = 0.680

*Note*: ^a^Significant with a Bonferroni corrected *p‐*value = 0.01.

## DISCUSSION

This study describes the co‐design process in which a shared goal‐setting tool was developed that we named ‘ENGAGE’. Co‐design partners were SLTs working with children with DLD and their parents in Dutch school settings or in SLT practices. We started with a list of items describing communicative participation of children with language disorders according to parents and professionals (Singer et al., 2020). We envisioned that a checklist of items would not be the best instrument to facilitate goal‐setting that is less therapist‐led. Instead, including SLTs perspective and needs and reflections from parents of children with DLD resulted in the co‐design of a physical artefact called ‘ENGAGE’, which we regarded to be more in line with family‐centred care and shared decision‐making. The tree‐like shape of the tool provides a positive metaphor for the growth and development of a child. Use of the tool allows the gradual introduction of items, and hence new information about the child's communicative functioning by both parents and the SLT. The tool supports the dialogue, shared decision‐making and goal‐setting process, and is flexible and intuitive in use. After several iterations performed in a usability study, the ratings on attractiveness, user‐friendliness and safety increased significantly, while the ratings for functionality and affordability remained at a satisfactory level.

From the role play session and the first ideating workshop it became apparent that the SLTs had a strong preference for developing a physical artefact. They expressed that this would serve their own needs and those of parents by facilitating dialogue and interaction. This result corresponds with the observation of Elwyn et al. (2010) that in face‐to‐face encounters sharing an artefact encourages dialogue because it typically requires both patient and clinician to shift body position and fix their gaze on the same information.

Some advantages of using co‐design are a better fit between an innovation and the user's needs, a better user experience, and higher user satisfaction (Steen et al., 2011). In this project, SLT–practitioners, SLT–researchers, and co‐design researchers were equally involved in the creative thinking and design process. Involving SLT–practitioners had the advantage that understanding how they feel, think, and act in the context of goal‐setting with parents provided insights in how to develop and optimize prototypes that would meet their needs (Sleeswijk Visser et al., 2005). We started with needs and wishes related to the organization of the content of the tool (i.e., the items) and during the project this focus shifted towards ideas and needs related to the format and functionality of the tool (i.e., how the items can be used to stimulate dialogue and interaction).

A key component of co‐design is that it builds on unique and individual experiences but it includes collaboration and collective perspectives too. Only eight SLT–practitioners participated in the first three stages. This low number of participants constitutes a risk of developing a solution that is not recognized as such by the larger group of end users. To test this, we invited new groups of SLT–practitioners to rate and usability test the prototypes. Their positive ratings and feedback confirmed the usability of the prototype and implies that the impact of the solution can reach beyond those who are directly involved (Boyd et al., 2012).

A strength of our project is that the co‐design researchers who prepared and conducted the co‐design workshops were also the designers of the tool. Usually, a co‐design researcher's involvement ends after the ‘Discover’ and ‘Define’ stages. Insights gained from these stages would typically be used to brief another designer, who would then develop prototypes. In this project however, the ‘Develop’ and ‘Deliver’ stages were integral parts of the process, mainly because we had limited funding and therefore limited time available to reach a practicable end‐result. The ‘Develop’ and ‘Deliver’ stages were therefore conducted without hiring an external design studio. Instead, the co‐design researchers assumed a different role, which led to a ‘designer understanding phase’ that was much more elaborate than a traditional briefing could be (Sleeswijk Visser et al., 2007). Their involvement from the beginning of the project ensured a deep understanding of the problem to be solved, the functionality to be delivered, and the design and user requirements to be met.

A risk associated with having the same (co‐)designers involved throughout a project is that it may invite unwanted or preliminary control from the co‐design researchers towards a particular solution. However, we do not think this occurred because the co‐design researchers were asked to start design no earlier than at the end of the Discover and Define stages. Another risk might be that the intensive interaction between co‐design researchers and participants leads to positive of negative bias towards the ideas and input of one or more participants. In this case, we do not think that this happened, because the input was regularly reviewed and discussed with all participants. In addition, bias may still be translated into the content of the briefing for an external designer. Furthermore, from the perspective of the SLT–researchers, having the same co‐design researchers involved in all the stages in the design development process proved to be extremely efficient for the project; the first prototype was already highly usable and needed only minor revisions during the ‘Deliver’ stage when it was tested in a real care setting. This observation is in accordance with Steen et al. (2011) who conclude that co‐design helps to organize projects more efficiently.

Challenges in collaboration across research disciplines were discussed in two evaluation sessions with the co‐design and SLT–researchers, halfway and at the end of the project. We observed differences in research language and traditions between health sciences and design researchers. For example: SLT–researchers used the term ‘prototype’, referring to the prefinal version of the product, while the co‐design researchers thought of a tangible version of an early design idea. Similarly, SLT–researchers expected structured agendas and protocolled activities within co‐design workshops, whereas the co‐design research group allowed for flexibility in the choice for specific creative techniques. In addition, timelines and deadlines within the project were perceived differently between the two research groups. The SLT–researchers were focused on the end‐product and tried to direct the project towards a tangible product. The co‐design researchers, on the other hand, tended to focus on the insights gained from the workshops, and refrained from skipping stages or jumping to conclusions. While both research groups acknowledged that there were marked differences between the research traditions, both agreed that SLT–practitioners proved to be excellent candidates for participation in a co‐design project, because they easily understood co‐design techniques and participated fully in the different creative workshops. When working as a cross‐disciplinary team we think it is important to address differences in approaches and expectations openly, preferably both before the start and during the project (Stickdorn et al., 2018). This way, co‐design can help to improve interdisciplinary cooperation within organizations, which has been described as one of its benefits for organizations (Steen et al., 2011).

In addition to the challenges identified in our evaluation sessions, previous studies mention several other potential problems when using co‐design methodology, such as lack of project management skills, and difficulties in establishing, building, and maintaining relationships with many different stakeholders (Groenevelt et al., 2019). In the present project, we think that these risks were minimized because the co‐design researchers were experienced in the collaboration with allied health professionals, while the SLT–researchers had previous experience conducting research with SLT–practitioners.

This study adds to the increasing number of initiatives that use co‐design in the development of health care interventions. With this paper, we wanted to provide an example of a co‐design development process in the field of SLT. The detailed description of the process may give the reader insight in what a co‐design process entails and what the distinct roles are of the actors involved. We hope that other researchers in the field of SLT can benefit from this example when they wish to develop new products together with end‐users, whether they are patients or professionals. Methods and co‐design techniques are dependent on the specific problem addressed and the stakeholders involved. It is therefore important to note that the example as outlined in this paper should be seen as a source of inspiration only, rather than as a procedure or methodology.

## LIMITATIONS AND SUGGESTIONS FOR FURTHER RESEARCH

A limitation in this study lies in the fact that SLT–practitioners were not directly involved in the actual selection of one of the three prototypes. Instead, the research team evaluated the three prototypes against the design guidelines and usability criteria that were developed with the SLT–practitioners. Including SLT–practitioners directly might have yielded different insights, or even a different solution. We chose not to do this, because the three prototypes had diverse levels of detail and sophistication, which may have influenced SLT–practitioners’ decision. When evaluating the project, we felt that including SLT–practitioners in the selection process would have been more appropriate because it again brings different perspectives together. However, this would only have been possible if all prototypes had the same level of detail, which required additional time from the designers. This limitation highlights the importance of carefully considering which decisions at what point in the process are made jointly or by subgroups only, and to plan the project accordingly. Another limitation is that we did not inquire if and how participation in the project changed SLT–practitioners’ perspectives on shared goal‐setting and collaboration with parents.

A significant limitation of our co‐design process is that parents’ input on the development of tool was not sought. Our focus was on SLTs because we felt that the SLTs held the key to change of their own behaviour in service delivery, and hence to the change from therapist‐directed to shared goal‐setting. While the tool sets out to enhance a dialogue between parent and SLT, parents’ involvement was limited to an interview on their children's wellbeing and values in life via participating SLT–practitioners, and in the usability testing. In retrospect, we should have included parents as equal partners in the co‐design process. Based on experiences in other projects where parents are part of the research team, we are now convinced that parents could have made make a valuable contribution in any co‐process aimed to improve the care for their children. Including the parents’ perspective in therapy is an essential component of evidence‐based practice (E3BP, Dollaghan, 2007). Similarly, parents’ participation in a co‐design process can be very empowering and can break down barriers to participate in society (Sleeswijk Visser et al., 2005), while it can also be challenging to involve non‐professionals in a design process (Groeneveld et al., 2019). However, more systematic research is needed that evaluates patients’ actual experiences of the co‐design activities (Bombard et al., 2018). ENGAGE was developed for use with parents of young children (aged 2–7 years) with (or at risk for) DLD, which is the typical age when children are identified in the Netherlands (Wiefferink et al., 2020). These children are too young to participate a co‐design process with written instruction and communication, but we think that there is an urgent need to develop tools and methods for shared goal‐setting with children that incorporates their unique perspectives, aspirations, and challenges. Methodologies that are tailored to engage (young) children in research, for example, through drawing, are increasingly being developed, tested and applied in SLT (e.g., Holliday et al., 2009).

A final limitation is that our description of the co‐design process ends with testing of several prototypes. Two additional steps must be taken before a tool is ready to be used in clinical practice: valorization and implementation. Future research could focus on how co‐design can help with the implementation of project results. In addition, research is needed on how SLTs, parents and children experience use of the tool, and how shared goal‐setting with a tool like ENGAGE impacts on therapy outcomes.

## CONCLUSIONS

The co‐design approach resulted in a shared decision‐making tool that was quite different from a traditional pen‐and‐paper questionnaire or test. Inclusion of the needs, experiences, and perspectives of SLTs in each stage of the development process resulted in a physical artefact that we named ENGAGE. The tool is aimed at supporting shared goal‐setting with parents, and also providing a positive metaphor for the growth and development of a child. Our project is an example of co‐design research with SLT end‐users. We hope that inclusion of professionals, but also children or adults with communication disorders and their families, will become best practice in the development of new tools, instruments and interventions for speech and language therapy.

## CONFLICT OF INTEREST

The authors report no conflicts of interest. The authors alone are responsible for the content and writing of the paper. All authors read, edited and contributed to the manuscript.

## Data Availability

Data are available upon request from the authors.
